# Inhibition of Prolyl Oligopeptidase Prevents Consequences of Reperfusion following Intestinal Ischemia

**DOI:** 10.3390/biomedicines9101354

**Published:** 2021-09-29

**Authors:** Alessia Filippone, Giovanna Casili, Alessio Ardizzone, Marika Lanza, Deborah Mannino, Irene Paterniti, Emanuela Esposito, Michela Campolo

**Affiliations:** Department of Chemical, Biological, Pharmaceutical and Environmental Sciences, University of Messina, Viale Stagno d’Alcontres, 98166 Messina, Italy; afilippone@unime.it (A.F.); gcasili@unime.it (G.C.); aleardizzone@unime.it (A.A.); mlanza@unime.it (M.L.); deborah.mannino@unime.it (D.M.); ipaterniti@unime.it (I.P.); campolom@unime.it (M.C.)

**Keywords:** intestinal ischemia/reperfusion injury, inflammation, angiogenesis, intestinal barrier permeability, apoptosis

## Abstract

Background: Intestinal ischemia/reperfusion injury (IRI) remains a clinical event that contributes to high morbidity and mortality rates. Intestinal epithelium is exposed to histological and vascular changes following tissue ischemia. Prolyl endopeptidase (PREP), involved in inflammatory responses, could be targeted for recovery from the permanent consequences following intestinal ischemia. Our aim was to investigate the role of PREP inhibitor KYP-2047 in tissue damage, angiogenesis, and endothelial barrier permeability after intestinal IRI in mice. Methods: KYP-2047 treatments were performed 5 min prior to intestinal damage. Intestinal IRI was induced in mice by clamping the superior mesenteric artery and the celiac trunk for 30 min, followed by 1 h of reperfusion. Results: PREP inhibition by KYP-2047 treatment reduced intestinal IR-induced histological damage and neutrophil accumulation, limiting inflammation through decrease of NF-ĸB nuclear translocation and fibrotic processes. KYP-2047 treatment restored barrier permeability and structural alteration following intestinal IRI, attenuating neovascular processes compromised by ischemia/reperfusion. Additionally, loss of epithelial cells during intestinal ischemia occurring by apoptosis was limited by KYP-2047 treatment, which showed strong effects counteracting apoptosis and DNA damage. Conclusions: These findings provide the first evidence that PREP inhibition through KYP-2047 inhibitor use could be a validate strategy for resolving alterations of intestinal epithelium the pathophysiology of intestinal disease.

## 1. Introduction

Mesenteric ischemia (MI) is a severe pathological event associated with a varied number of diseases such as vessel obstruction, hernias, and septic shock often resulting in morbidity and death [1]. MI essentially leads to developing acute and chronic inflammatory conditions of the intestine and in the worst case developing intestinal ischemia (II) [2]. The II refers to a complex consequence including loss of organ-specific activity, alteration of gut microbiota, tissue necrosis, and the release of proinflammatory cytokines (interleukin 1β [IL-1β] and tumor necrosis factor α [TNF-α]) and reactive oxygen species (ROS). This cascade is driven by the nuclear factor nuclear factor kappa-light-chain-enhancer of activated B cells (NF-κB) which promotes inflammatory responses and dependent in situ recruitment of neutrophils, macrophages, and immune cells. Increased local damage and systemic inflammation following II is amplified with reperfusion, resulting in cell and tissue injuries associated with an uncontrolled anaerobic products release, generation of numerous pro-inflammatory cytokines, and the activation of immune cells [3]. Intestinal ischemia/reperfusion injury (IRI) occurs as a result of many clinical conditions and reperfusion can magnify the damage, and if local and systemic inflammation remain uncontrolled, they can induce remote multisystem organ failure (MSOF) and dysfunctions. Although the mechanisms of intestinal IRI are complex and still not well-understood, changes in vascular permeability and mucosal barrier integrity are deeply correlated. Particularly, following ischemia, the intestinal mucosa is exposed to long-lasting changes in its architecture and integrity translating to an accumulation of collagen in the intestinal wall and alterations in tight junctions (TJs) distribution along with intestinal neighbored epithelial cells [4]. Furthermore, an intestinal injury may lead to severe damage in blood vessel mechanisms such as alterations in neoangiogenic and pre-existing vasculature processes, critical steps for a multitude of biological functions [5]. Since II injury has been studied considerably as a cause of subsequent intestinal epithelial cell death through necrosis, it is understandable to assume that the apoptotic event is recognized as the active and predicted event following ischemia. Recent studies reported that the intestine is prone to triggering apoptotic stimulation of p53 protein, the most studied tumor suppressor, following a restricted period of blood flow interruption, which directs transcriptional control of extrinsic and intrinsic pathways together with correlated promoters and modulators [6]. Although it has been established that intestinal functionality and barrier integrity are importantly compromised following intestinal ischemia/reperfusion, few studies to date have explored a therapeutic target for defending subsequent altered processes. Prolyl endopeptidase (PREP) is a serine protease with enzymatic activity and the ability to cleave short proline-containing peptides (smaller than 3 kDa) involved in inflammatory response activation, neurodegenerative diseases development, and autoimmune diseases’ outcome. Moreover, has been reported that PREP inhibitor treatment gives beneficial effects for cognitive disorders [7]. In the present study, we aimed to investigate the effects of PREP inhibition through the small-molecule KYP-2047 inhibitor used to fight angiogenesis and apoptosis in a mouse model of intestinal IRI.

## 2. Materials and Methods

### 2.1. Animals

Male adult CD1 mice, 6 weeks old (25–30 g, Envigo, Udine, Italy), were housed in a controlled environment and provided with standard rodent chow and water, in stainless steel cages in a room kept at 22 ± 1 °C with a 12 h light, 12 h dark cycle. The animals were acclimatized to their environment for 1 week, having ad libitum access to tap water and standard rodent diet. This study was approved by the University of Messina Review Board for the care of animals, in compliance with Italian regulations on protection of animals (n 399/2019-PR released on 24 May 2019). Animal care was in accordance with Italian regulations on the use of animals for experiments (D.M. 116192) as well as with EEC regulations (O.J. of E.C. L 358/1 12/18/1986).

### 2.2. Surgical Procedure for Intestinal IRI

Male mice were allowed access to food and water ad libitum, and a midline laparotomy was performed. The celiac and superior mesenteric arteries were isolated near their aortic origins maintaining intestinal tract warmed. IRI was induced by clamping the superior mesenteric artery and the celiac trunk, resulting in a total occlusion of these arteries for 30 min. After this period of occlusion, the clamps were removed, and the splanchnic circulation was allowed reperfusion for 1 h, following a splenic artery occlusion (SAO) procedure. After that reperfusion, animals were killed, and each ileum tract was isolated and used for further analysis [8]. Mice in the KYP-2047 treatments groups were subjected to the intraperitoneal (i.p.) administration (doses of 1, 2.5, and 5 mg/kg) 5 min before the reperfusion. Briefly, we penetrated the lower left quadrant of the abdomen 5 mm deep in a line parallel with the backbone and at a 45° angle to the abdominal wall [9,10,11]. At starting, mice were randomly divided into five groups (10 mice in each group): sham group; IRI + vehicle group; IRI + KYP-2047 1 mg/kg group; IRI + KYP-2047 2.5 mg/kg group, and IRI + KYP-2047 5 mg/kg group.

### 2.3. Experimental Groups

Mice were randomly divided into the following groups:

- Sham group: mice were operated on with surgical steps; however, they were not subjected to IR and were treated with either a vehicle (saline).
- IRI + vehicle group: mice (*n* = 10) were subjected to intestinal ischemia by SAO (30 min), followed by reperfusion (1 h);
- IRI + KYP-2047 1 mg/kg group: mice (*n* = 10) were subjected to surgical procedures, described as above, and KYP-2047 (1 mg/kg, i.p.) was administered 5 min prior to reperfusion);
- IRI + KYP-2047 2.5 mg/kg group: mice (*n* = 10) were subjected to surgical procedures described as above, and KYP-2047 (2.5 mg/kg i.p.) was administered 5 min prior to reperfusion);
- IRI+ KYP-2047 5 mg/kg group: mice (*n* = 10) were subjected to surgical procedures described as above, and KYP-2047 (5 mg/kg i.p.) was administered 5 min prior to reperfusion).

To determine KYP-2047 specificity for blocking PREP, the expression level of PREP was measured in a pilot experiment in mice (*n* = 4) following different doses’ administration (1, 2.5, and 5 mg/kg) at 1 h 30 min. Moreover, since I/R + KYP-2047 1 mg/kg group histological evaluation did not reveal any significant beneficial effects compared to the I/R group, we decided to keep KYP-2047 2.5 mg/kg and 5 mg/kg doses for all further analysis.

### 2.4. Histological Examination

Ileum tissues were collected after 1 h of reperfusion and stained as previously described [12]. The morphological criteria were considered as already described [8]. The degree of intestinal damage was evaluated according on a six-point scale: 0 = no inflammation, 1 = mild inflammation, 2 = mild/moderate inflammation, 3 = moderate inflammation, 4 = moderate/severe inflammation and 5 = severe inflammation. Sections were also stained by Masson’s Trichrome method for collagen detection and observed under light microscopy (Zeiss Microscope, Axiostar Plus, Milan, Italy). The degree of fibrosis was evaluated as % fibrotic area (blue staining) and quantified using image analysis software (Image J 1.8.0).

### 2.5. Myeloperoxidase Activity (MPO)

MPO activity, which is an indicator of polymorphonuclear leukocyte (PMN) accumulation, was determined spectrophotometrically at 650 nm [13]. MPO activity was expressed in U per gram weight of wet tissue and defined as the quantity of enzyme degrading 1 μmol of peroxide min^−1^ at 37 °C.

### 2.6. Western Blot Analysis for PREP, NF-κB Pathway, Angiogenesis and Apoptosis Markers

Western blots were performed as described in our previous study [14]. Specific primary antibodies were used: nuclear factor kappa-light-chain-enhancer of activated B cells (NF-κB) (1:500, sc 8008, Santa Cruz Biotechnology, Dallas, TX, USA) or nuclear factor of kappa light polypeptide gene enhancer in B-cells inhibitor, alpha (IκB-α, 1:500, sc 1643, Santa Cruz Biotechnology, Dallas, TX, USA), or anti-ciclooxigenase 2 (COX-2) (1:500, sc 1746, Santa Cruz Biotechnology, Dallas, TX, USA) or prolyl endopeptidase (PREP) (1:500, sc 365416, Santa Cruz Biotechnology, Dallas, TX, USA), or anti-inducible nitric oxide synthetase (iNOS) (1:500, sc 8310, Santa Cruz Biotechnology, Dallas, TX, USA), or Bax (1:500, sc 7480, Santa Cruz Biotechnology, Dallas, TX, USA), or Bcl-2 (1:500, sc 7382, Santa Cruz Biotechnology, Dallas, TX, USA), or VEGF (1:500 sc 7269, Santa Cruz Biotechnology, Dallas, TX, USA), or eNOS (1:500 sc-654, Santa Cruz Biotechnology, Dallas, TX, USA). The stripping and re-probing of the WB membrane was performed. Lastly, to ascertain if those blots were loaded with equal amounts of proteins, they were also incubated in the presence of the antibody against β-actin (cytosolic fraction 1:500; Santa Cruz Biotechnology, TX, USA) or lamin A/C (nuclear fraction 1:500, MilliporeSigma, Burlington, MA, USA) as previously described [14].

### 2.7. Immunofluorescence (IF) for Zonula Occludens (ZO)-1 and Filaggrin (FLG)

Immunofluorescence staining was performed as previously described [15]. The following antibodies were used: murine monoclonal anti-zona occludens-1 (ZO-1) (1:100, sc 8147, Santa Cruz Biotechnology, Dallas, TX, USA) or mouse monoclonal anti-filaggrin (FLG) (1:100, sc 80609, Santa Cruz Biotechnology, Dallas, TX, USA).

### 2.8. Immunohistochemical Localization of Vascular Endothelial Growth Factor (VEGF) and CD34

The immunohistochemical staining was executed as previously described by Casili G and colleagues [16]. The following primaries were used: monoclonal anti-VEGF antibody (1:100, sc 7269, Santa Cruz Biotechnology, Dallas, TX, USA) and polyclonal anti-CD34 polyclonal antibody (1:100, sc 74499, Santa Cruz Biotechnology, Dallas, TX, USA).

### 2.9. TUNEL Assay

Apoptosis showed a peak one hour after reperfusion, as detected in the nuclei of cells by the TUNEL method. TUNEL assay was conducted by using a TUNEL detection kit according to the manufacturer’s instructions (Apo-Tag, HRP kit; DBA, Milan, Italy) and as previously described [17].

## 3. Results

### 3.1. Inhibition of PREP by KYP-2047 Reduces Intestinal IR-Induced Histological Damage and Neutrophil Accumulation in the Intestine

**Figure 1:**
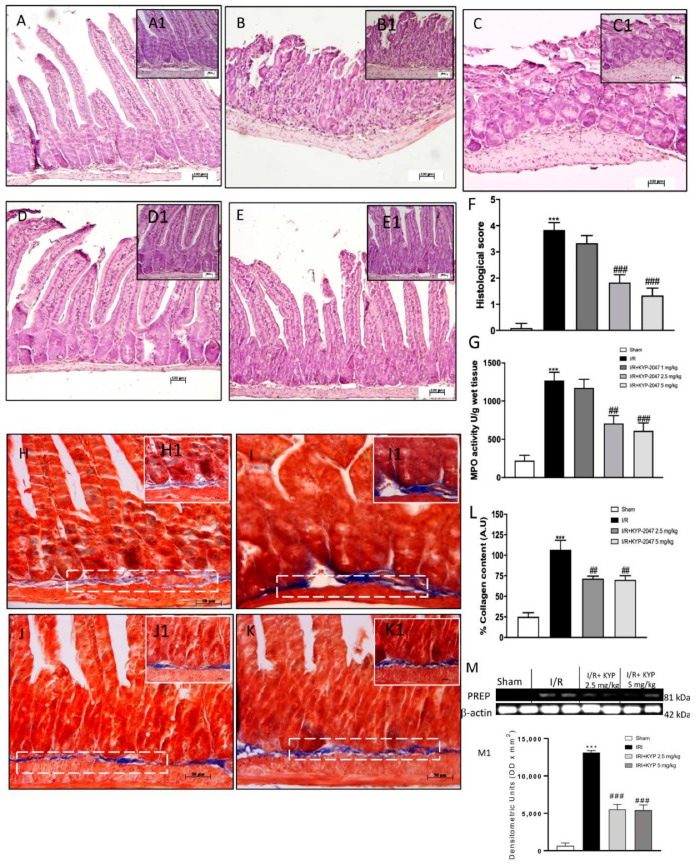
Effect of KYP-2047 on histological damage of intestinal tissue, MPO activity and collagen detection. (**A**) No histological damage was observed in control group. (**B**) Intestinal IRI group showed a significant alteration of the mucosal architecture. (**C**) KYP-2047 1 mg/kg did not show significant reduction of tissue damage. (**D**) KYP-2047 2.5 mg/kg treatment repair of damaged tissue following intestinal IRI. (**E**) KYP-2047 2.5 and 5 mg/kg treatments showed a significant restoration of the architecture of the intestinal mucosa. (**F**) Histological score. (**G**) Effect of KYP-2047 on the MPO activity. Neutrophil accumulation, was observed in the IRI group. KYP-2047 2.5 mg/kg and 5 mg/kg treatments significantly reduced MPO activity. (**H**) No fibrosis was detected in control group. (**I**) IRI group showed a significant increase in fibrosis compared to the control group (blue area). (**J**,**K**) The degree of fibrosis in the KYP-2047 2.5 mg/kg and 5 mg/kg treated groups was significantly reduced compared to IRI group. (**L**) Quantification of collagen content (%). (**M**) Expression levels evaluation of PREP in the intestine samples. (M1) Densitometric analysis referred to blot in panel M. Data are representative of at least three independent experiments. Data is expressed as mean ± S.E.M. *** *p* < 0.001 vs. Sham; ### *p* < 0.001, ## *p* < 0.01 vs. IRI.

IRI is typically accompanied by histological features such as shortening of the villi, loss of villous epithelium, and general tissue architecture [18]. Histopathological analyses of intestines confirmed that the intestinal IRI group (Figure 1 panel B, magnifications B1) resulted in significant mucosal injury compared to the sham group (Figure 1 panel A, magnifications A1). However, alterations of mucosal architecture IR-induced were significantly repaired by KYP-2047 treatment especially at the higher doses of 2.5 mg/kg and 5 mg/kg (Figure 1D,E, magnification D1 and E1, and see densitometric analysis panel F). MPO activity, an indicator of neutrophil accumulation, was also evaluated. Compared to sham, an increase in MPO activity was found in IR tissue (Figure 1, panel G), and treatment with KYP-2057 1 mg/kg did not restore intestinal damage. Then, we found that IRI-induced recruitment of neutrophils was significantly reduced in KYP-2047 treated animals, indicating that KYP-2047 treatments (2.5 mg/kg and 5 mg/kg) was able to mitigate intestinal inflammation (Figure 1G).

### 3.2. KYP-2047 Administration Led to Limit Progressive Intestinal Inflammation and Fibrosis following IRI

Extracellular matrix (ECM) localization produced by an expanded fibroblast activation is a consequence of intestinal inflammation and fibrosis [19]. Masson trichrome staining revealed that, in the IR-injured intestine (Figure 1I, magnification I1) an increase in fibrosis (blue-stained area) was significantly reported compared to the non-inflamed intestine (Figure 1H, magnification H1, see percent fibrotic area panel L), whereas in KYP-2047 2.5 mg/kg and 5 mg/kg treated animals (Figure 1J,K, magnification J1 and K1), the degree of fibrosis was smaller than the injured intestine (see percent fibrotic area Figure 1L). Moreover, it is known that KYP-2047 is a selective inhibitor of PREP (Ki = 23 pM) that inhibits about 85% PREP activity within 10 min of administration [16a]; thus, to clearly demonstrate the specific inhibition of PREP by KYP-2047, we performed Western blot analysis in intestine samples. A basal expression level of PREP was observed in samples from the control group and compared to the significant increase observed in intestinal IRI group (Figure 1M). The higher doses of KYP-2047 (2.5 and 5 mg/kg) were shown to be effective in terms of inhibiting PREP expression in the intestine, whereas no changes were found for the doses of 1 mg/kg (Figure 1M, see densitometric analysis M1). 

### 3.3. Inhibition of PREP Attenuated the NF-κB Pathway Activation following Intestinal IRI

**Figure 2:**
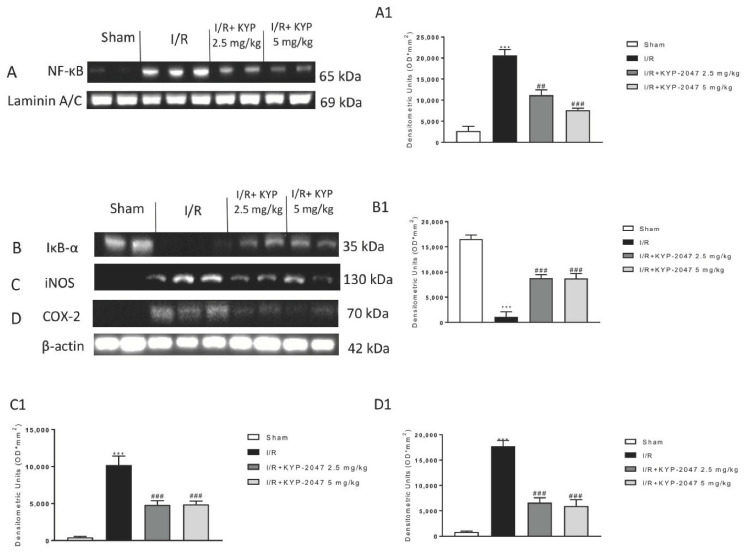
Effects of KYP-2047 on NF-ĸB pathway and pro-inflammatory enzymes iNOS and COX-2. (**A**) Western blot analysis revealed that in the IRI group there was a significant increase of NF-ĸB levels compared to the sham group and a significant decrease in IĸB-α expression levels. (**A1**) Densitometric analysis referred to blot in panel A. (**B**) KYP-2047 2.5 mg/kg and 5 mg/kg treated groups showed a significant decrease of NF-ĸB nuclear translocation and a significant increase of IĸB-α levels. (**B1**) Densitometric analysis referred to blot in panel B. (**C**,**D**) Expression levels of pro-inflammatory iNOS and COX-2 enzymes were increased in IRI group compared to the sham group, while a significant decrease was observed in the KYP-2047 2.5 mg/kg and 5 mg/kg treated groups. (**C1**,**D1**) Densitometric analysis referred to blots in panels C and D. Data is expressed as mean ± S.E.M. Data are representative of at least three independent experiments. *** *p* < 0.001 vs. Sham; ## *p* < 0.01 vs. IRI; ### *p* < 0.001 vs. IRI.

The pro-inflammatory factors that are released following NF-κB activated pathway as interleukins and other mediators are expressed from intestinal epithelial cells and stimulate the immune cells of the intestine epithelial monolayer in response to ischemic stimuli [20]. Western blot analysis revealed an increase in NF-κB nuclear translocation along with a decrease of IκB−α degradation following IRI as well as increased iNOS and COX-2 expression levels when compared to the sham group (Figure 2A–D, see densitometric analysis A1, B1, C1, and D1). Pharmacological inhibition of PREP by KYP-2047 proved that intraperitoneal administration at the doses of 2.5 mg/kg and 5 mg/kg decreased translocation of NF-κB and I IκB−α degradation, significantly exhibiting a reduced inflammatory response activation (Figure 2A,B, see densitometric analysis A1 and B1). Moreover, the levels of iNOS and COX-2 protein were markedly repressed by PREP inhibition (Figure 2C,D, see densitometric analysis C1 and D1). These results revealed that suppression of PREP by KYP-2047 efficiently decrease intestinal pro-inflammatory molecules’ production, preventing NF-κB inflammatory cascade activation.

### 3.4. Treatment with KYP-2047 Stabilized the Expression of Tight Junction Zona Occludens-1 (ZO-1) and Restored Structural and Functional Role of Filaggrin (FLG) in the Intestine

**Figure 3:**
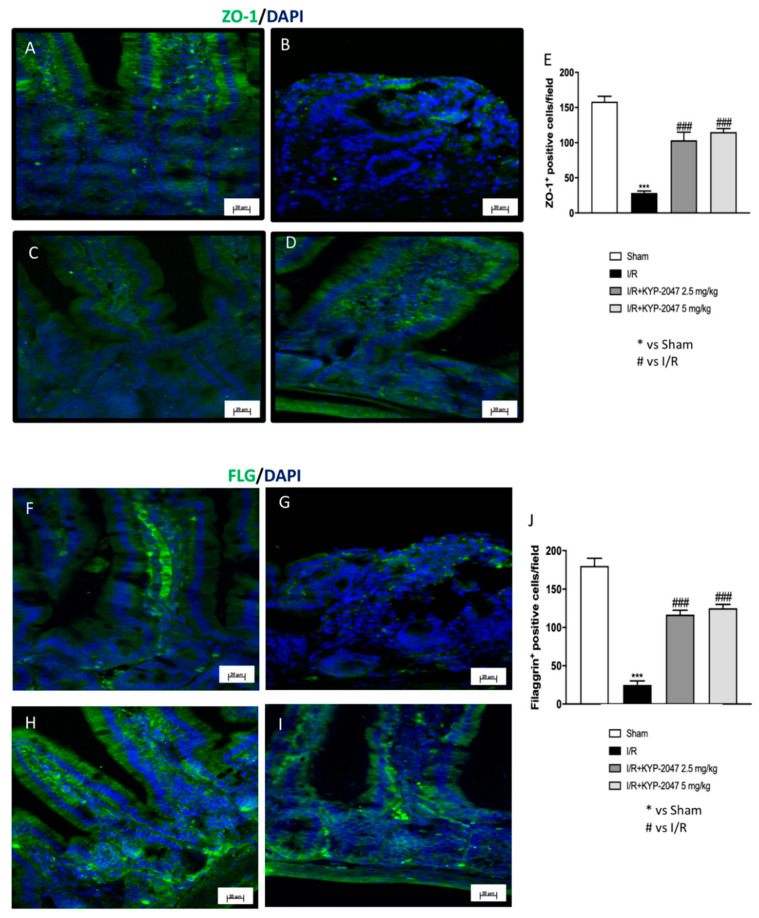
Effect of KYP-2047 administration on the expression level of ZO-1 and FLG by IF in intestine. (**A**) Control group intestinal cells’ immunoreactivity for ZO-1. (**B**) IRI animals showed a significant loss of intestinal ZO-1. (**C**,**D**) An increase in ZO-1 cells’ positivity was observed in IRI animals and subsequently treated with KYP-2047 (2.5 mg/kg and 5 mg/kg). (**E**) Immunofluorescence analysis of ZO-1. (**F**) The number of positive FLG cells was normal in the sham group. (**G**) A significant decrease in FLG cells’ positivity was observed in IRI animals. (**H**,**I**) KYP-2047 treatments (2.5 mg/kg and 5 mg/kg) increased FLG cells’ positivity in the intestine. (**J**) Immunofluorescence analysis of FLG. Data are representative of at least three independent experiments. *** *p* < 0.001 vs. Sham; ### *p* < 0.001 vs. IRI.

To morphologically investigate the intestinal barrier permeability lost following intestinal IRI IF, ZO-1 and FLG were performed on ileum sections. In control animals (Figure 3A), ZO-1 (green) distribution can be observed in the paracellular junctions, while, in IRI animals, there was a significant loss of intestinal ZO-1 IF positive staining (Figure 3B). However, intestinal IRI inducing loss of barrier integrity was restored by KYP-2047 treatments (2.5 mg/kg and 5 mg/kg) (Figure 3C,D, see analysis panel E). This is followed by an increase in ZO-1 cells positivity in KYP-2047-treated animals, suggesting that mucosal barrier integrity collapse may be repaired by PREP inhibition to attenuate I/R damage. Another important marker of intestinal barrier dysfunction is FLG [21]. A lower FLG immunoreactivity in the intestine from intestinal IRI mice was revealed compared to sham animals (Figure 3G,F, respectively). The numbers of FLG-immunolabeled cells were increased in KYP-2047-treated animals, highlighting an important role in the restoration of intestinal barrier integrity (Figure 3H,I, see analysis panel J). 

### 3.5. VEGF-Mediated Vascular Patterning and the Neovascular Process Are Regulated by KYP-2047 during IRI

**Figure 4:**
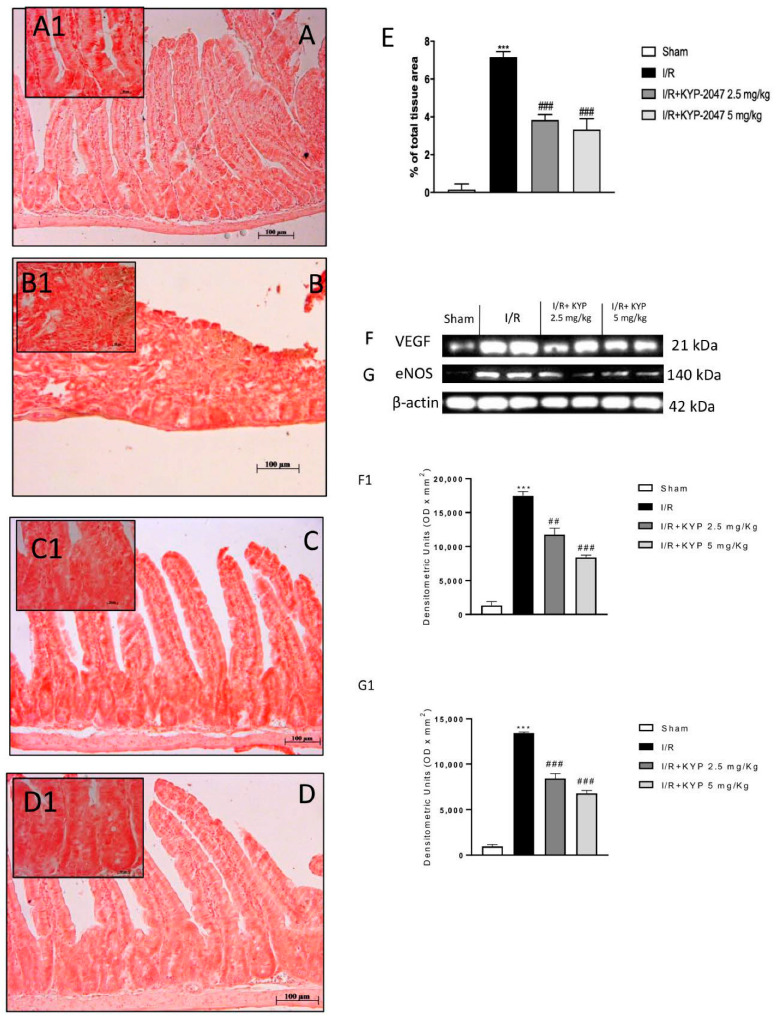
Effect of KYP-2047 on angiogenesis pathway. (**A**) No VEGF immunoreactivity was showed in control intestines. (**A1**) 20 × magnification panel A. (**B**) Increased VEGF cell positivity was observed in IRI intestine. (**B1**) 20 × magnification panel B. (**C**,**D**) The degree of positive staining for VEGF was markedly reduced in tissue sections after KYP-2047 2.5 mg/kg and 5 mg/kg treatments. (**C1** and **D1**) 20 × magnification panel C and D, respectively (**E**) Western blot analysis for VEGF. (**F**) Western blot analysis for eNOS showed increased expression levels in the IRI group. KYP-2047 2.5 mg/kg and 5 mg/kg treatments restored of eNOS levels was observed. (**F1**) Densitometric analysis referred to blot in panel F. (**G**,**G1**). Data are representative of at least three independent experiments. One-way ANOVA test *** *p* < 0.001 vs. sham; ## *p* < 0.01 vs. IRI; ### *p* < 0.001 vs. IRI.

Since VEGF is not only a strong developer of angiogenesis, but also increases vascular permeability and is up-regulated during hypoxia [22], the effect of KYP-2047 on VEGF signaling was further investigated. VEGF angiogenic switch during intestinal-IRI was evaluated by immunostaining in the intestine; therefore, it was identified in the lamina propria of the ileum mucosa. VEGF staining in the ileum villi of the sham group was basal in comparison with intestinal IRI animals that showed an increase in the cell positivity (Figure 4A,B, magnification A1 and B1, see densitometry analysis panel E). As shown in Figure 4, the up-regulation of VEGF induced by intestinal IRI was reduced following the administration of KYP-2047 at the doses of 2.5 and 5 mg/kg (Figure 4C,D, magnification C1 and D1). In addition, western blot analysis showed that intestinal-IRI significantly promoted VEGF expression levels in the intestine when compared to the control group (Figure 4F, see densitometry analysis F1). However, KYP-2047 treatment at the doses of 2.5 mg/kg and 5 mg/kg significantly reversed the VEGF levels to close to control levels (Figure 4F, see densitometry analysis F1).

VEGF has been shown to regulate NO production; therefore, it is reasonable that NO levels were regulated by increased eNOS expression, a marker significantly increased during intestinal ischemia [23]. Our data showed that KYP-2047 treatments attenuated eNOS expression following intestinal IRI (Figure 4G, see densitometry analysis G1). Thus, our results revealed that PREP inhibition driven by KYP-2047 administration suppressed the angiogenic process via VEGF/eNOS modulation, influencing the earliest stages of vascular development induced by intestinal ischemia-reperfusion. 

**Figure 5:**
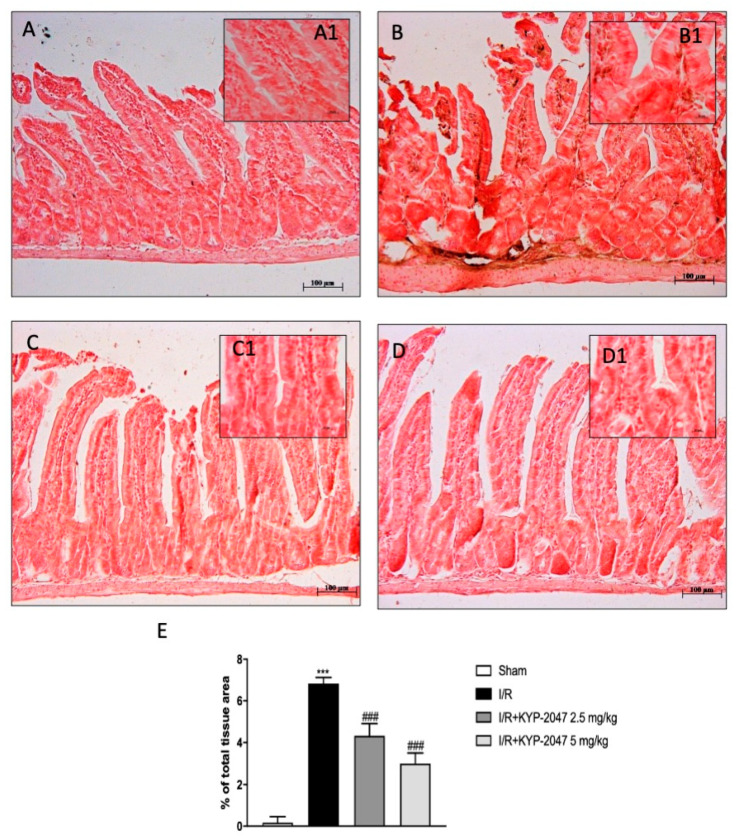
Effect of KYP-2047 treatments on CD34 expression in the tunica mucosa of the intestine. (**A**) No CD34 immunoreactivity was shown in control intestines. (**A1**) 20 × magnification panel A. (**B**) CD34 immunoreactivity was increased after IRI. (**B1**) 20 × magnification panel A. (**C**,**D**) CD34 immunoreactivity was decreased in the KYP-2047 groups (2.5 mg/kg and 5 mg/kg). (**C1,D1**) 20 × magnification panel C and D, respectively. (**E**) Immunohistochemical analysis of CD34. Data are representative of at least three independent experiments. *** *p* < 0.001 vs. sham; ### *p* < 0.001 vs. IRI.

Moreover, CD34, a glycoprotein expressed on the cell membrane of endothelial cells of small blood vessels, was used as a novel marker for vascular endothelial cells with a focus on intestinal cells following intestinal IRI [24]. CD34-positive cells were found in the tunica mucosa of the ileum [25]. A significant amount of the total CD34-positive cells in intestinal tissue from the intestinal IRI group compared to sham tissues was detected (Figure 5A,B, magnification B1 and A1, respectively). KYP-2047 treatment following intestinal ischemia, at both 2.5 and 5 mg/kg doses, showed a significant decreased of immunopositivity for CD34 (Figure 5C,D, magnification C1 and D1, see densitometry analysis panel E). This evidence, which supports an essential role for angiogenic factors in intestinal IRI, suggests that both the VEGF/eNOS pathway and CD34 modulation are required for efficient vascular endothelial differentiation, giving reasonable use of KYP-2047 in attenuation of vascular alterations in intestinal diseases. 

### 3.6. Apoptosis Effects of KYP-2047 on the Bcl-2/Bax Pathway and DNA Fragmentation following Intestinal IRI

**Figure 6:**
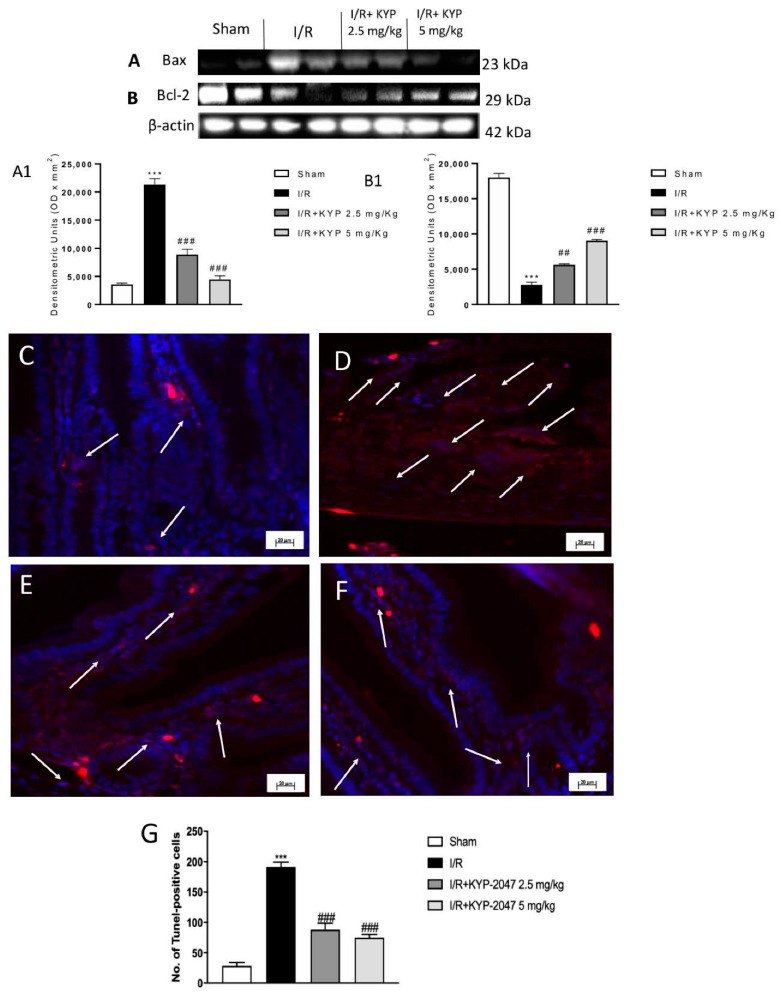
Effect of KYP-2047 treatment on apoptosis pathway. (**A**) Western blot analysis revealed increased expression levels in Bax after IRI. Expression levels of Bax were significantly reduced by KYP-2047 treatment (2.5 mg/kg and 5 mg/kg). *** *p* < 0.001 vs. sham; ### *p* < 0.001 vs. IRI. (**A1**) Densitometry analysis referred to panel A. (**B**) Western blot analysis showed decreased in Bcl-2 expression levels after IRI compared to the control group. KYP-2047 (2.5 mg/Kg and 5 mg/Kg) treatments significantly restored that reduction. (**B1**) Densitometry analysis referred to panel B. (**C**) Few TUNEL-positive apoptotic cells were found in control mice, compared with an increase in TUNEL-positive cells on IRI mice (**D**–**F**) KYP-20147 treatments significantly reduced the rate of cell apoptosis. (**G**) Quantification of TUNEL staining. Data are representative of at least three independent experiments. Each data is expressed as mean ± SEM from *** *p* < 0.001 vs. sham; ### *p* < 0.001 vs. IRI; ## *p* < 0.01 vs. IRI.

Loss of epithelial cells during intestinal ischemia occurs by apoptosis, firstly in crypts and then in adjacent uninvolved areas [26]. Bax and Bcl-2 could play a key role; thus, we evaluated their expression by western blot analysis. In the IRI intestine, the Bax expression level was found to be up-regulated, suggesting a potent activation of apoptosis compared to intestine from control animals. In comparison, intestinal IRI-induced Bax expression was significantly attenuated by KYP-2047 treatments at both doses (2.5 and 5 mg/kg) (Figure 6A, see densitometry analysis A1) suggesting that regulation of PREP could reflect in apoptosis mitigation. Contrarily, accompanied changes in apoptosis of the intestinal environment following intestinal IRI were confirmed by obtaining low levels of Bcl-2 (Figure 6B, see densitometry analysis B1). Therefore, intestinal cell death was reduced following KYP-2047 administration, confirming that apoptosis arrest occurs via PREP inhibition.

To further examine the role of apoptosis in IRI-induced intestinal injury and the protective effects of KYP-2047, we analyzed TUNEL-stained intestine sections. Intestinal IRI caused an increase in intestinal apoptosis as shown by the high number of TUNEL-positive cells compared to the sham group (Figure 6C,D, respectively) whereas PREP inhibition by KYP-2047 had significant effects on apoptosis, as evidenced by a decrease in the number of TUNEL-positive intestinal cells (Figure 6E–G). Taken together, these data indicated that KYP-2047 administration attenuated apoptosis and its involved targets.

## 4. Discussion

Intestinal IRI is a significant exponent of the awfulness and mortality related to mesenteric artery occlusion, aortic manipulation, and hemorrhagic shock [27]. These complex forms of ischemic injury include well-defined signs of the ischemic event as well as excessive interstitial pressure, which probably lead to epithelial sloughing (Huang 2011). Despite the vast number of experimental findings reporting that controlled cell death is critical to the maintenance of intestinal barrier functionality and epithelial homeostasis [28], there is only limited information on the rapid response and renewal of injured intestinal tissue to blood flow arrest. In this study, we presented important discoveries demonstrating for the first time that a selective inhibition of PREP by KYP-2047 reduces intestinal damage, restores mucosal barrier function, and reduces the angiogenesis as well as apoptosis processes in an in vivo model of IRI. Our laboratory already demonstrated that KYP-2047 was efficient in preventing the activation of inflammatory pathways and influencing angiogenesis events [29].

In this study, we revealed that the intestinal damage following IRI led to disruption of villous apical surface and damage in deeper mucosa and submucosa layers. Histological observation revealed that the intestinal tissue injury was significantly regenerated and remodeled in mice subjected to KYP-2047 treatment, indicating that PREP inhibition was able to well recover histological alteration even following an elevated degree of intestinal injury associated with reperfusion. Similarly, neutrophil infiltration was increased by IRI and reversed similarly to control levels with KYP-2047 treatment, explaining its protective effects against ischemia-reperfusion damage. Previous studies reported that varied causes lead to the compromise of organ activity and even intestinal failure following I/R injury. For instance, the excessive accumulation of ECM (extracellular matrix) is a common event that occurs in various intestinal pathologies including ulcerative colitis, intestinal bowel, and Crohn’s disease [30]. The increase of ECM in the intestine, with collagens and fibronectins deposition into the mucosa, submucosa, muscularis mucosa, muscularis propria, and serosa layers, can lead to development of intestinal architecture alterations and obstructions. This is because the key event of intestinal fibrosis is not only inflammatory cells’ recruitment but also fibroblast and myofibroblast differentiation and ROS production that can activate mesenchymal and non-mesenchymal cells, leading to fibrotic tissue formation [31]. Our results clearly showed that PREP inhibition reduced intestinal fibrosis in mice, demonstrating that KYP-2047 administration may contribute to preventing intestinal fibrosis and reducing ECM deposition following intestinal IRI. 

During ischemic conditions, the prompt release of certain pro-inflammatory cytokines and correlated molecules has been supposed to contribute significantly to intestinal mucosal dysfunction through the NF-κB pathway [32]. We confirmed the ability of KYP-2047 to reduce the NF-κB nuclear translocation and Iκb-α degradation according to a decreased release of pro-inflammatory mediators including iNOS and COX-2, emphasizing the role of PREP inhibitor to reduce the inflammation process in intestinal IRI [33]. The decreased immunoreactivity of intestinal cells to essential proteins correlated to the maintenance of intestinal barrier integrity such as ZO-1 and FLG could be correlated with the intestinal IRI-induced unbalanced intestinal homeostasis. Therefore, we found an increase in the number of positive cells for ZO-1 and FLG when mice were subjected to KYP-2047 oral administration, highlighting that PREP inhibition not only was able to arrest inflammatory pathway activation but also attenuated and restored the loss of intestinal barrier functionality to normal conditions.

It is obvious to assume that an inadequate blood flow that occurs following an ischemic event is reflected in an unbalanced process of angiogenesis and, although associated regulators have been studied during intestinal injury, none were found to be efficacious. Angiogenesis is an essential process in all types of wound healing, including intestinal mucosal healing, and it is regulated by many proangiogenic factors, including VEGF, fibroblast growth factor (FGF), and endothelial growth factor (EGF) [34,35]. In a similar fashion, it has been suggested that injured intestinal cells produce nitric oxide (NO) during the reperfusion period, initiating and contributing apoptosis and vascular changes, and, in particular, promoting eNOS activation [36]. In our study, we used 2.5 and 5 mg/kg doses of KYP-2047 that were effective in protecting the angiogenesis process clearly altered by intestinal IRI. Both VEGF and eNOS’s expression levels were significantly reduced when mice were treated with either KYP-2047 dose. Moreover, since CD34, a cell-to-cell adhesion factor, is selectively expressed by vascular endothelial cells and participates in the angiogenic process, it was evaluated following intestinal IRI as a consistent player in vascular recovery. Our findings, suggested first that PREP inhibition could be useful to reduce an exaggerated new blood vessels generation during intestinal diseases through VEGF/eNOS attenuation, and second that increased cells’ positivity of CD34, recognized as a key regulator of neovascularization process, seemed to be attenuated in mice treated with both doses (2.5 and 5 mg/kg) of KYP-2047.

Moreover, 60 min of reperfusion after ischemia (30 min) was enough to demonstrate that the vascular and mucosal barrier has been damaged or lost because of a deregulated inflammatory response and aggravated cell death via apoptosis, a mechanism closely related [37]. As shown above, intensified NF-κB nuclear translocation, in turn, lead to increased expression levels of two of the main related-apoptotic effectors such as Bax, which possesses a pro-apoptotic role, and Bcl-2, a well-known anti-apoptotic player [38]. In our study, we found that activated apoptosis was significantly attenuated following KYP-2047 treatments, amplifying the concept that PREP inhibition allowed intestinal cells to recover their cellular equilibrium and counteracting apoptosis following ischemic injury.

## 5. Conclusions

In conclusion, this study has reported for the first time that KYP-2047, which is an important PREP negative modulator, could be a prominent pharmacological target in preventing most of the consequences of intestinal IRI, including intestinal fibrosis and barrier integrity alteration, inflammatory process, angiogenesis, and apoptosis, offering a new approach in preventing the exacerbation and progression of intestinal diseases.

## Data Availability

Not applicable.
